# Supplementary material for: Molecular Epidemiology and Genetic Diversity of Human Respiratory Syncytial Virus in Sicily during Pre- and Post-COVID-19 Surveillance Seasons
Source: Pathogens. 2023 Aug 28;12(9):1099. doi: 10.3390/pathogens12091099 (PMC10534943; doi:10.3390/pathogens12091099)
Supplement: Supplementary file 1 [file pathogens-12-01099-s001.zip › Table S1.pdf]

Table S1. Amino acid changes identified in the Sicilian dataset of hRSV-A protein G gene sequences and relative frequency. AA substitution and position are defined in comparison with the ON1 prototype strain ON67-1210A (GenBank accession number: JN257693).

Position	Substitution	Frequency (%)
**7**	Q --> L/R	2.7
**9**	T --> A	2.7
**26**	I --> T	0.9
**57**	A --> V	5.4
**67**	H --> N/Q	3.1
**69**	V --> F	0.8
**71**	L --> P	2.3
**78**	D --> N	0.8
**80**	T --> A/M	7.0
**82**	Q --> R	0.8
**86**	T --> I	0.8
**93**	Q --> L/R	1.6
**98**	G --> E	0.8
**99**	I --> T	1.6
**100**	S --> N	1.6
**101**	F --> L/S	5.4
**102**	S --> F/T	5.4
**106**	G --> E	4.6
**107**	T --> A	0.8
**108**	T --> I	0.8
**111**	S --> F/P	13.2
**113**	T --> I	7.7
**114**	I --> T	0.8
**115**	L --> P	3.1
**120**	P --> L	0.8
**128**	S --> P/T	9.3
**130**	T --> I	1.6
**131**	V --> I/D	8.5
**132**	K --> E	0.8
**133**	I --> V/T	4.6
**134**	K --> I	34.1
**135**	N --> K/T	3.1
**137**	T --> A	1.6
**140**	Q --> R	0.8
**142**	L --> S	13.2
**144**	S --> I	0.8
**146**	P --> L/S/T	22.5
**147**	T --> I	0.8
**149**	K --> R	0.8
**151**	R --> H	7.0
**152**	Q --> K	0.8
**154**	K --> R	0.8
**161**	N --> D	0.8
**163**	F --> L	0.8
**173**	C --> W	0.8
**178**	N --> D/G	8.5
**193**	K --> R	0.8
**200**	T --> I/P	2.3
**206**	P --> Q	20.2
**209**	K --> R	7.0
**210**	T --> I/S	2.3
**214**	D --> E	1.6
**215**	P --> L/S	3.1
**216**	K --> N/R	5.4
**222**	P --> L	2.3
**224**	E --> G/K/V	13.9
**225**	V --> A	9.3
**226**	L --> F/P	2.3
**227**	T --> I	2.3
**230**	P --> T	5.4
**231**	T --> A	0.8
**232**	G --> R	5.4
**234**	P --> L	7.0
**235**	T --> A	0.8
**239**	T --> A	0.8
**240**	K --> R/E	0.8
**241**	T --> P	0.8
**243**	I --> S	33.3
**245**	T --> A	0.8
**247**	L --> P/Q	8.5
**248**	L --> I	20.9
**249**	T --> N	0.8
**250**	S --> F	4.6
**253**	K --> R	8.5
**254**	G --> E/R	1.6

Position	Substitution	Frequency (%)
**255**	N --> D/S	3.1
**256**	P --> S	3.1
**257**	E --> K	3.1
**258**	H --> Q/Y	20.9
**262**	E --> K	34.1
**263**	E --> G/K	8.5
**265**	L --> F/I	6.2
**266**	H --> L	7.7
**267**	S --> L	3.1
**270**	S --> F/P/Y	3.9
**271**	E --> D/K	7.0
**272**	G --> S	2.3
**273**	Y --> H/S	7.7
**274**	L --> P	58.1
**275**	S --> G/N	3.1
**276**	P --> Q/S	2.3
**278**	Q --> L	2.3
**279**	V --> I	1.6
**280**	Y --> H	3.1
**284**	G--> D/S	10.1
**285**	Q --> R	4.6
**287**	E --> D/G/K	2.3
**289**	L --> P	5.5
**291**	S --> L	11.7
**294**	S --> P/Y	2.3
**295**	E --> G/K/V	3.9
**296**	G --> S	6.2
**298**	L --> P/S	77.3
**299**	S --> I	0.8
**300**	P --> S	3.1
**303**	V --> A/I	21.9
**304**	Y --> H	57.0
**306**	T --> I	1.6
**308**	E --> K	2.3
**310**	L --> P	29.5
**311**	S --> P	7.0
**313**	S --> F	3.9
**314**	L --> P	25.6
**317**	S --> F	6.2
**319**	T --> A/I/S	13.2
**320**	T --> A/I/K	34.1
