# Supplementary material for: Molecular Epidemiology and Genetic Diversity of Human Respiratory Syncytial Virus in Sicily during Pre- and Post-COVID-19 Surveillance Seasons
Source: Pathogens. 2023 Aug 28;12(9):1099. doi: 10.3390/pathogens12091099 (PMC10534943; doi:10.3390/pathogens12091099)
Supplement: Supplementary file 1 [file pathogens-12-01099-s001.zip › Table S2.pdf]

Table S2. Amino acid changes identified in the Sicilian dataset of hRSV-A protein G gene sequences, according to the surveillance season. Within each season, cells shaded by pin color mean codon changes observed at low frequency (30% - 50%), whereas cells highlighted by violet color refer to codon changes observed at high frequency (>50%) Deduced amino acid changes and position are defined in comparison with the ON1 prototype strain ON67-1210A (GenBank accession number: JN257693).

hRSV-A		SURVEILLANCE SEASON							
AA position		2015/16	2016/17	2017/18	2018/19	2019/20	2020/21	2021/22	2022/23
7	Q			R	L				
9	T			A					
26	I	T							
57	A							V	V
67	H	N			Q	N			
69	V					F			
71	L	P				P			
78	D	N							
80	T		A						M
82	Q							R	
86	T				I				
93	Q	L							
98	G		E						
99	I	T							
100	S	N							
101	F					L		S	S
102	S	F/T	F			F			
106	G		E		E				
107	T			A					
108	T					I			
111	S	P	F			P			
113	T				I	I		I	I
114	I			T					
115	L	P	P						
120	P								L
128	S		P/T						
130	T		I		I				
131	V				D	D/I		D	D
132	K	E							
133	I		T			V			
134	K	I	I	I	I	I			
135	N		K					T	
137	T			A		A			

hRSV-A		SURVEILLANCE SEASON							
AA position		2015/16	2016/17	2017/18	2018/19	2019/20	2020/21	2021/22	2022/23
140	Q								R
142	L		S		S	S			S
144	S					I			
146	P	T				S		L	S
147	T		I						
149	K				R				
151	R	H			H			H	H
152	Q								K
154	K	R							
161	N					D			
163	F		L						
173	C	W							
178	N		D		G	G		G	G
193	K		R						
200	T			I/P	P				
206	P		Q	Q		Q		Q	
209	K		R			R		R	R
210	T		I			S			
214	D		E						
215	P			L	L/S				
216	K	N/R	N		N	N			
222	P	L							L
224	E	G			K	V			V
225	V	A	A		A			A	A
226	L			F		P		F	
227	T			I					
230	P	T						T	T
231	T	A							
232	G		R		R				
234	P					L			L
235	T					A			
239	T		A						
240	K					R			E
241	T		P						
243	I	S	S	S	S	S			
245	T								A
247	L	Q			P	P			P
248	L		I	I		I		I	I

hRSV-A		SURVEILLANCE SEASON							
AA position		2015/16	2016/17	2017/18	2018/19	2019/20	2020/21	2021/22	2022/23
249	T				N				
250	S							F	F
253	K		R						
254	G					R			E
255	N			D	D/S				
256	P		S						
257	E	K							
258	H		Y		Q	Q		Q	Q
262	E	K	K	K	K	K			
263	E	G				G		K	K
265	L	I			F	F			
266	H				L	L		L	L
267	S			L					L
270	S	F	Y			P			
271	E	K	K		D	K			K
272	G				S				S
273	Y	S	H	H	H	H			H
274	L	P	P	P	P	P		P	P
275	S		G/N	N					
276	P	S				Q			
278	Q	L							
279	V			I	I				
280	Y	H		H		H			H
284	G		D/S						D
285	Q		R						
287	E		G/K	K		D			
289	L		P		P				
291	S					L			
294	S		Y			P			
295	E	K	K	V	G				
296	G		S						S
298	L	P	P	P	P	P		P	P
299	S	I							
300	P	S	S						
303	V		A	A	I	A		A	A
304	Y		H	H	H	H		H	H
306	T		I						
308	E	K	K						

hRSV-A		SURVEILLANCE SEASON							
AA position		2015/16	2016/17	2017/18	2018/19	2019/20	2020/21	2021/22	2022/23
310	L	P	P	P	P	P		P	P
311	S					P			P
313	S							F	
314	L	P	P			P			P
317	S								F
319	T	A	I	I		S		I	I
320	T	I	A/K	A	A	A		A	A
