# Supplementary material for: Molecular Epidemiology and Genetic Diversity of Human Respiratory Syncytial Virus in Sicily during Pre- and Post-COVID-19 Surveillance Seasons
Source: Pathogens. 2023 Aug 28;12(9):1099. doi: 10.3390/pathogens12091099 (PMC10534943; doi:10.3390/pathogens12091099)
Supplement: Supplementary file 1 [file pathogens-12-01099-s001.zip › Table S3.pdf]

Table S3. Amino acid changes identified in the Sicilian dataset of hRSV-B protein G gene sequences and relative frequency. AA substitution and position are defined in comparison with the BA9 prototype strain BA4128/99B (GenBank accession number: AY333364).

Position	Substitution	Frequency (%)
**5**	K --> R	1.0
**6**	N --> S	1.0
**18**	D --> N	2.0
**57**	A --> T	1.2
**69**	V --> I	0.6
**74**	V --> I	0.6
**76**	V --> I	4.0
**87**	I --> M/T	3.5
**90**	Y --> H	0.6
**91**	L --> P	8.6
**94**	V --> I	0.6
**97**	E --> D	0.6
**98**	R --> K	1.1
**99**	V --> A	0.6
**100**	S --> G/N	10.3
**102**	S --> P	0.6
**105**	L --> P	100.0
**106**	T --> I	0.5
**107**	T --> A/D	99.4
**108**	T --> A/P	2.3
**109**	P --> Q/S	2.3
**110**	P --> L/S	2.3
**112**	Y --> H/Q	99.4
**113**	T --> I/M	1.0
**115**	S --> L/P	4.0
**116**	A --> T	2.3
**117**	T --> A	1.1
**118**	I --> T	3.4
**120**	P --> T	2.3
**121**	N --> T	1.4
**122**	T --> A	0.6
**124**	S --> L	2.3
**126**	T --> A	1.4
**128**	H --> Y	0.6
**129**	T --> K	0.6
**130**	T --> I	17.6
**131**	A --> T	23.3
**133**	T --> A	1.1

Position	Substitution	Frequency (%)
**135**	G --> D/S	2.8
**136**	R --> I/T	99.4
**137**	T --> I	22.2
**138**	T --> S	100.0
**140**	P --> L/S	7.9
**142**	Q --> H/K	2.8
**143**	N --> K/S	1.1
**144**	N --> H	0.6
**149**	K --> E	0.6
**152**	P --> S	0.6
**154**	N --> K	4.5
**156**	P --> S	1.1
**157**	K --> *	100.0
**159**	K --> *	100.0
**171**	V --> I	0.5
**174**	S --> G	0.5
**175**	I --> V	1.1
**177**	G --> S	0.5
**192**	N --> S	0.5
**194**	P --> Q	2.2
**199**	T --> A	0.5
**200**	I --> T	100.0
**204**	N --> K/T	1.7
**205**	K --> Q	0.5
**206**	P --> S	1.1
**207**	P --> T	0.5
**209**	K --> E	0.5
**211**	T --> K	3.9
**212**	N --> T	0.5
**213**	K --> E	0.5
**214**	R --> K	1.1
**215**	D --> H/Y	3.3
**216**	P --> L/S	12.1
**217**	K --> I	0.5
**218**	K --> P/T	100.0
**219**	L --> P	100.0
**220**	A --> S	0.5
**222**	T --> P	0.5

Position	Substitution	Frequency (%)
**223**	L --> M/P/S/T	89.5
**224**	K --> E	0.5
**225**	K --> E	1.1
**227**	T --> A/N	19.3
**228**	T --> A	1.7
**229**	I --> T	7.7
**231**	P --> L/S	5.5
**232**	T --> A	0.5
**233**	K --> R	0.5
**234**	K --> N	0.5
**236**	T --> I	0.5
**237**	P --> L/S	2.2
**239**	T --> I	0.5
**240**	T --> K	1.7
**243**	D --> G/N	3.9
**244**	T --> S	0.5
**245**	S --> N	1.7
**247**	S --> P	98.3
**249**	S --> P	2.2
**251**	V --> A/M	2.2
**252**	L --> F/P	2.2
**254**	T --> I	73.5
**255**	T --> N	0.5
**256**	T --> I/S	2.2
**257**	S --> P	2.8
**258**	K --> N/R	9.4
**259**	H --> Q	0.5
**260**	T --> K	0.5
**261**	E --> K	1.1
**263**	D --> E/N/Y	2.2
**267**	S --> L/P	12.7
**269**	S --> F	1.7
**270**	T --> A/I	79.0
**271**	V --> A	91.7
**272**	L --> F/P	9.4
**277**	S --> P	8.3
**281**	I --> T	99.4
**282**	Q --> L	1.1
**284**	Q --> L	2.2
**285**	S --> F/P	1.7
**286**	L --> P	5.0
**287**	H --> Y	76.8

Position	Substitution	Frequency (%)
**290**	T --> I	39.2
**291**	P --> S	1.7
**292**	E --> K/Q	1.1
**296**	N --> S/F	1.1
**297**	S --> M	0.5
**302**	T --> A/I	1.1
**303**	A --> P/T	10.5
**304**	S --> F	2.2
**305**	E --> D/K	3.9
**306**	P --> S	0.5
**307**	S --> P/Y	1.1
**308**	T --> I	0.5
**309**	S --> P	0.5
**312**	T --> A/F/I/N	32.6
**313**	Q --> *	84.0
**314**	K --> R	87.3
**316**	* --> Q	92.8
**317**	S --> P	1.1
**318**	Y --> C	0.5
**322**	F --> I/S	1.1
