# Supplementary material for: Molecular Epidemiology and Genetic Diversity of Human Respiratory Syncytial Virus in Sicily during Pre- and Post-COVID-19 Surveillance Seasons
Source: Pathogens. 2023 Aug 28;12(9):1099. doi: 10.3390/pathogens12091099 (PMC10534943; doi:10.3390/pathogens12091099)
Supplement: Supplementary file 1 [file pathogens-12-01099-s001.zip › Table S4.pdf]

Table S4. Amino acid changes identified in the Sicilian dataset of hRSV-B protein G gene sequences, according to the surveillance season. Within each season, cells shaded by pin color mean codon changes observed at low frequency (30% - 50%), whereas cells highlighted by violet color refer to codon changes observed at high frequency (>50%) Deduced amino acid changes and position are defined in comparison with the BA9 prototype strain BA4128/99B (GenBank accession number: AY333364).

HRSV-B		SURVEILLANCE SEASON							
AA position		2015/16	2016/17	2017/18	2018/19	2019/20	2020/21	2021/22	2022/23
5	K				S				
6	N								R
18	D				N				
57	A								T
69	V	I							
74	V	I							
76	V	I	I						
87	I	T	M						
90	Y	H							
91	L	F/P	F						
94	V				I				
97	R					D			
98	R				K				
99	V	A							
100	S	N						G	G
102	S		P						
105	L	P	P	P	P	P		P	P
106	T								I
107	T	A	A/D	A	A	A		A	A
108	T	P							A
109	P	S			S				Q/S
110	P		L/S						
112	Y	H	H	H/Q	H	H		H	H
113	T		M		I				
115	S	L		P					
116	A		T			T		T	
117	T	A							
118	I		T						
120	P	T							
121	N				T				
122	T	A							
124	S							L	
126	T		A		A				

HRSV-B		SURVEILLANCE SEASON							
AA position		2015/16	2016/17	2017/18	2018/19	2019/20	2020/21	2021/22	2022/23
128	H				Y				
129	T					K			
130	T	I							I
131	A			T	T	T		T	
133	T								A
135	G	D	S						S
136	R	T	I/T	T	T	T		T	T
137	T			I	I	I		I	
138	T	S	S	S	S	S		S	S
140	P		S			L			
142	Q	H	K						
143	N	S			K				
144	N				H				
149	K		E						
152	P	S							
154	N	K	K		K				
156	P	S	S						
157	K	*	*	*	*	*		*	*
159	K	*	*	*	*	*		*	*
171	V		I						
174	S					G			
175	I	V							
177	G					S			
192	N		S						
194	P			Q					
199	T								A
200	I	T	T	T	T	T		T	T
204	N	T	K/T						
205	K		Q						
206	P				S				
207	P	T							
209	K	E							
211	T							K	
212	N		T						
213	K	E							
214	R	K							
215	D		H	Y					
216	P	L	S					S	S

HRSV-B		SURVEILLANCE SEASON							
AA position		2015/16	2016/17	2017/18	2018/19	2019/20	2020/21	2021/22	2022/23
217	K	I							
218	K	T	T	T	T	T		T	P/T
219	L	P	P	P	P				
220	A		S						
222	T		P						
223	L	M/P/T	P/S/T	P	P	P/S		P	
224	K	E							
225	K	E							
227	T	N	N		A/N				N
228	T	A		A	A				
229	I		T		T			T	
231	P	S		L	L				
232	T								A
233	K		R						
234	K			N					
236	T				I				
237	P		S	L	L				
239	T		I						
240	T	K	K						
243	D	N		G					
244	T					S			
245	S		N						
247	S	P	P	P	P	P		P	P
249	S	P							
251	V	A	A	M					
252	L		F/P						
254	T	I	I	I	I	I			
255	T			N					
256	T		I	S	S				
257	S			P					
258	K		N/R		N			N	N
259	H		Q						
260	T								K
261	E	K	K						
263	D		E/Y						N
267	S	P		L/P	P			P	
269	S	F	F						
270	T	I	A/I	I	I	I			

HRSV-B		SURVEILLANCE SEASON							
AA position		2015/16	2016/17	2017/18	2018/19	2019/20	2020/21	2021/22	2022/23
271	V	A	A	A	A	A		A	A
272	L	F/P		P				P	
277	S							P	P
281	I	T	T	T	T	T		T	T
282	Q								L
284	Q			L					
285	S	F/P	P						
286	L		P	P					
287	H	Y	Y	Y	Y	Y		Y	
290	T		I	I	I	I		I	I
291	P	S							
292	E				K			Q	
296	N			F					S
297	S		M						
302	T				I				A
303	A	T	P/T	P					
304	S			F					
305	E		D	K				K	K
306	P	S							
307	S		Y		P				
308	T		I						
309	S		P						
312	T	N	A	I	I	I/F		I	I
313	Q	*	*	*	*	*		*	*
314	K	R	R	R	R	R		R	
316	*	Q	Q	Q	Q	Q		Q	Q
317	S	P							
318	Y				C				
322	F	S	I						
